# Successful Treatment of Noise-Induced Hearing Loss by Mesenchymal Stromal Cells: An RNAseq Analysis of Protective/Repair Pathways

**DOI:** 10.3389/fncel.2021.656930

**Published:** 2021-11-23

**Authors:** Athanasia Warnecke, Jennifer Harre, Matthew Shew, Adam J. Mellott, Igor Majewski, Martin Durisin, Hinrich Staecker

**Affiliations:** ^1^Clinic for Otolaryngology–Head & Neck Surgery, Hanover Medical School, Hanover, Germany; ^2^Cluster of Excellence “Hearing4all” of the German Research Foundation (EXC 2177/1), Oldenburg, Germany; ^3^Department of Otolaryngology–Head & Neck Surgery, Washington University School of Medicine in St. Louis, St. Louis, MO, United States; ^4^Ronawk Inc., Olathe, KS, United States; ^5^Department of Otolaryngology–Head & Neck Surgery, University of Kansas School of Medicine, Kansas City, KS, United States

**Keywords:** noise trauma, hearing loss, mesenchymal stroma cells, Wharton’s jelly, cochlear transcriptome, hearing protection

## Abstract

Mesenchymal stromal cells (MSCs) are an adult derived stem cell-like population that has been shown to mediate repair in a wide range of degenerative disorders. The protective effects of MSCs are mainly mediated by the release of growth factors and cytokines thereby modulating the diseased environment and the immune system. Within the inner ear, MSCs have been shown protective against tissue damage induced by sound and a variety of ototoxins. To better understand the mechanism of action of MSCs in the inner ear, mice were exposed to narrow band noise. After exposure, MSCs derived from human umbilical cord Wharton’s jelly were injected into the perilymph. Controls consisted of mice exposed to sound trauma only. Forty-eight hours post-cell delivery, total RNA was extracted from the cochlea and RNAseq performed to evaluate the gene expression induced by the cell therapy. Changes in gene expression were grouped together based on gene ontology classification. A separate cohort of animals was treated in a similar fashion and allowed to survive for 2 weeks post-cell therapy and hearing outcomes determined. Treatment with MSCs after severe sound trauma induced a moderate hearing protective effect. MSC treatment resulted in an up-regulation of genes related to immune modulation, hypoxia response, mitochondrial function and regulation of apoptosis. There was a down-regulation of genes related to synaptic remodeling, calcium homeostasis and the extracellular matrix. Application of MSCs may provide a novel approach to treating sound trauma induced hearing loss and may aid in the identification of novel strategies to protect hearing.

## Introduction

Sound trauma can lead to hearing loss with protean manifestations. With exposures ranging from acute blast injury to chronic noise exposure, a wide range of pathological effects are induced in the cochlea. The degree of injury that occurs is related to the energy and frequency of the input sound combined with the time of exposure (Le et al., 2017). At higher levels of sound exposure, damage to stereocilia and loss of hair cells in a basal to apical gradient occurs resulting in a permanent threshold shift. Damage to the spiral ganglion can occur through excitotoxicity, inflammation and loss of trophic support (Chen et al., 1997; Puel et al., 1997; Lin et al., 2011). Additionally, severe sound trauma results in degradation of the blood labyrinthine barrier. Sound trauma therefore causes a range of cellular damage that includes both hair cells and spiral ganglion neurons as well as changes in the cochlear blood supply. The molecular mechanisms involved in these processes have been extensively investigated. Initial sound overexposure results in oxidative stress with overproduction of reactive oxygen and nitrogen species leading to hair cell lipid membranes peroxidation and disruption of mitochondrial homeostasis (Yamane et al., 1995). The mitochondrion has also been implicated as a primary generator of free radicals and appears to be intimately involved in the generation sound trauma related pathophysiology (Kurabi et al., 2017). Multiple studies have shown that antioxidant defenses and the ability to buffer free calcium of the cells can prevent noise trauma (Huang et al., 2000; Gonzalez-Gonzalez, 2017). Ultimately, there is an activation of a variety of mitogen-activated protein (MAP) kinase stress pathways including c-Jun N-terminal (JNK) kinase leading to programmed cell death/apoptosis. Apoptosis can also be activated through tumor necrosis factor alpha (TNFα), which is produced during sound trauma (Kurabi et al., 2017). Other pro-inflammatory cytokines are produced in a delayed fashion via an nuclear factor kappa-light-chain-enhancer of activated B-cells (NFκB) signaling cascade (Kurabi et al., 2017). More recent studies have focused on erythroid two related factor 2 (Nrf2) being a key signal mediator at the center of multiple pathways (Fetoni et al., 2019). This molecule acts as a reactive oxygen species (ROS) sensor and normally exists in a bound state in the cytoplasm. It controls a variety of stress responses and events such as oxidative stress and inflammation can cause it to enter the nucleus and activate a variety of signaling cascades (Fetoni et al., 2019). Evaluation of gene expression networks after sound trauma has implicated several key pathways in sound trauma mainly relating to inflammation, heat shock response and detoxification of ROS (Clifford et al., 2016; Yang et al., 2016). Changes in regulation of genes related to cytokine signaling and pathways related to innate immunity appear to play an important role in the early response to sound trauma (Patel et al., 2013; Vethanayagam et al., 2016; Yang et al., 2016). Single cell RNAseq analysis after sound trauma demonstrates that a mix of adaptive and innate immune cells infiltrate the cochlea after sound trauma highlighting the complexity of the damage response (Rai et al., 2020). Antioxidants and anti-inflammatory drugs clearly can protect against noise induced hearing loss although, given the complexity of the induced reactome, it is unlikely that a single pharmaceutical agent would adequately protect against all aspects of damage (Rajguru, 2013; Maeda et al., 2017).

Many of the transcriptome changes seen in sound trauma are also seen in traumatic brain injury (TBI) and stroke, suggesting common mechanisms and potentially common approaches to attenuating these injuries (Meng et al., 2017; Li et al., 2020). An alternate approach to cochlear protection is treatment of inner ear injury with neuronal or mesenchymal stem cells/mesenchymal stromal cells (MSCs). MSCs have been demonstrated to have a wide range of immunomodulatory and protective effects after tissue injury and have been tested in diverse disorders such as stroke, acute kidney injury and myocardial infarction (Squillaro et al., 2016; Fu et al., 2017; Chang et al., 2018; Cunningham et al., 2018; Harrell et al., 2019; Tsintou et al., 2020). Fat derived MSCs have been shown to express growth factors in sound exposed inner ears (Fetoni et al., 2014). Both peripheral and local delivery of MSCs have been shown to have protective effects in a variety of hearing loss models but the molecular consequences of MSC delivery after sound trauma have not been evaluated [Reviewed in Warnecke et al. (2017); Kanzaki et al. (2020)].

Mesenchymal stromal cells have been postulated to work through two distinct mechanisms. When delivered into the peripheral circulation, MSCs can home into areas of damaged tissue and through a range of distinct signaling pathways including TGFβ, CCL2, IL6, IDO, VEGF, and WNT modulate inflammation and induce tissue protection and repair. It has been shown that in this delivery approach, the majority of injected MSCs are trapped in in the pulmonary circulation and are phagocytosed by pulmonary macrophages. This process of efferocytosis results in the release of factors inducing immune tolerance such as IL-10 and TGFβ, which may provide additional beneficial effects (Galipeau and Sensébé, 2018). Local delivery of MSCs also induces immunomodulatory and protective effects through direct delivery of growth factors and extracellular vesicles in their secretome. Some studies suggest that interaction between the MSCs and the damaged tissue is needed for the most complete protective effects (Wakabayashi et al., 2010; Drago et al., 2013; Hsieh et al., 2013). Local delivery of MSCs to the brain after TBI has been shown to partially normalize pathologic gene expression induced by trauma, resulting in a return to normal immune signaling, receptor mediated cell signaling, neuronal plasticity and glycolysis (Darkazalli et al., 2017). This suggests that cell therapy with MSCs has the capacity to provide multi-mechanistic protection and repair. To evaluate the mechanisms underlying the effects of MSCs on the inner ear, we treated adult mice exposed to an acute narrow band sound trauma with Wharton’s jelly-derived MSCs (WJCs) directly delivered into the inner ear. Umbilical cord-derived cells like WJCs, were chosen since they have been shown to have a secretome that supports neuronal repair (Hsieh et al., 2013). After treatment, changes in the cochlear transcriptome and in hearing threshold were evaluated. With this investigative approach, we identified up and down regulated genes in mice exposed to noise and treated with WJC when compared to mice exposed to noise trauma only.

## Materials and Methods

All animal procedures were approved by the Institutional Animal Care and Use Board. A total of 25 4 week old female C57BL/6 mice (Jackson Laboratories) weighing between 18 and 23 grams were utilized for the experiments described below.

### Procurement and Expansion of Mesenchymal Stromal Cells

Human MSCs were isolated from Wharton’s jelly of umbilical cords according to the protocols approved by the University of Kansas Human Subjects Committee (KU-Lawrence IRB approval #15402) and Stormont-Vail (SMV) Hospital (SMV IRB Approval conferred by IRB Chair, Jo-Ann S. Harris, MD). Informed consent was obtained from the patients before collection of the umbilical cords. Three umbilical cords (*n* = 3) were obtained from Stormont-Vail (Topeka, KS, United States). All cords were from males that were born at full term and delivered under normal delivery conditions. Human MSCs were isolated and cultured according to previously published protocols (Mellott et al., 2016). Cells were cultured in medium consisting of 1% penicillin-streptomycin (Life Technologies), 10% fetal bovine serum (FBS) mesenchymal stem cell qualified (MSCq) (Life Technologies), and fibroblast basal medium (Lonza Group Ltd., Basel, Switzerland), and expanded to passage 5 for all experiments. For delivery, cells were harvested, washed in phosphate buffered saline (PBS) and resuspended to a concentration of 1 × 10^6^ cells/ml in sterile PBS.

### Experimental Design

Three groups were evaluated with five animals in each experimental condition. Group one consisted of animals (*n* = 5) that underwent assessment of baseline hearing by auditory brainstem response (ABR) followed by MSC delivery into the left ear. The same animals underwent re-evaluation of hearing with ABR 7 days post-MSC delivery. Group two animals were exposed unilaterally to sound followed by treatment with MSC (*n* = 5) or artificial perilymph (*n* = 5) at 48 h post-sound trauma. These animals were sacrificed 72 h post-MSC delivery for extraction of RNA from the treated (left-sided) cochlea. Group 3 animals were unilaterally exposed to sound followed by MSC (*n* = 5) or artificial perilymph (*n* = 5) at 48 h post-sound trauma. These animals underwent hearing testing with ABR at 14 days post-cell or perilymph therapy to evaluate rescue of hearing and thereafter sacrificed for histological analysis. Group 4 animals were exposed unilaterally to sound followed by treatment with MSC (*n* = 8) or artificial perilymph (*n* = 8) at 48 h post-sound trauma. These animals were sacrificed 72 h post-MSC delivery for extraction of RNA from the treated (left-sided) cochlea (*n* = 5) or perfused for immunohistochemistry (*n* = 3). An additional set of age matched untreated control animals underwent extraction of RNA from the cochlea (*n* = 5) or were perfused for immunohistochemistry (*n* = 3).

### Auditory Brainstem Response Measurements

For ABR testing, the mice were anesthetized intraperitoneally using a mixture of ketamine (100 mg/kg) and xylazine (10 mg/kg). Mice were then placed inside a double-walled, sound attenuated chamber. Body temperature was maintained at 37°C using a MediHeat V500Vstat Heated Operating Table Digital Thermostat (PECO Services). ABR measurements were acquired using the Smart EP program from Intelligent Hearing Systems. Needle electrodes were placed on the vertex (+), behind the left ear (−) and behind the opposite ear (ground). Tone bursts were presented at 4, 8, 16, and 32 kHz, with duration of 500 μs using a high frequency transducer. Recording was carried out using a total gain equal to 100 K and using 100 Hz and 15 kHz settings for the high and low-pass filters. A minimum of 128 sweeps was presented at 90 dB sound pressure level (SPL). The SPL was decreased in 10 dB steps. Near the threshold level, 5 dB SPL steps using up to 1024 presentations were carried out at each frequency. Threshold was defined as the SPL at which at least one of the waves could be identified in 2 or more repetitions of the recording.

### Sound Exposure

For the noise trauma (sound exposure), the mice were anesthetized as described above. Mice were then exposed to a 16 kHz pure tone presented at 118 dB SPL in the left ear for 4 h. Sound was delivered through a speaker equipped with a ribbon tweeter (Radio Shack 40-1310 Horn Super Tweeter). The speaker was coupled to the left ear via a short plastic tube, 12 mm in inner diameter and 45 mm in length. Prior to exposure, the sound output was calibrated using a Quest Electronics Precision Integrating Sound Level Meter (model 1800). The Sound Level Meter was calibrated using a 1000 Hz Bruel & Kjaer 4230 Sound Level Calibrator. After sound exposure, animals were allowed to recover for 48 h before initiating treatment.

### Delivery of Mesenchymal Stromal Cells

Mice were anesthetized as described above. A dorsal post-auricular incision was made, and the left posterior semicircular canal exposed. Using a microdrill, a canalostomy was created, exposing the perilymphatic space. Subsequently 1 μl of MSCs (1 × 10^6^ cells/ml in sterile PBS) were injected using a Hamilton microsyringe with 0.1 μl graduations and a 36-gauge needle). Control animals received 1 μl injection of artificial perilymph into the canal. The canalostomy was sealed with bone wax. The mice showed no signs of vestibular dysfunction or head tilt post-operatively.

### RNA Extraction and Analysis

Group 2 animals (noise trauma plus MSC or perilymph treatment) were anesthetized with Beuthanasia 75 mg/kg (Schering-Plough Animal Health Corp., Union, NJ, Canada). The treated (left) cochlea was removed and immediately placed in RNAlater (Qiagen, cat #76104). Total RNA was extracted with Trizol reagent (Thermofisher, cat #15596018) and purified by centrifuging with phase lock heavy gel (Tiagen, cat # WMS-2302830). Concentration and quality of RNA were assessed using an Agilent Bioanalyzer 2100. Analysis of cochlear RNAs was carried out using the NuGen Universal Plus Assay on a NovaSeq sequencer as per manufacturer instructions. Trimming, quality control and alignment was carried out using the A.I.R. software package^1^ with noise damage only specimens as a reference and noise damage with MSC treatment as the analyzed data package. There were five replicates for each experimental condition. The C57Bl/6 mouse genome (C57Bl6NJvi/ENSEMBL release) was used as a reference genome. Statistical analysis was carried out by the NOISeq method. Up regulated and down regulated genes were identified and organized according to their gene ontology (GO) classification. A combination of the enrichment score and the false discovery rate (FDR) was then used to develop a gene ontology enrichment analysis (GOEA). Analysis of potential biological significance was carried out using Ingenuity Pathway Analysis (IPA) software (Qiagen Bioinformatics, Redwood City, CA, United States) and the Reactome Knowledgebase^2^ (Jassal et al., 2019). Using the IPA software package, results were organized into the most common pathways that were altered in response to MSC treatment and which keys genes were up or down regulated. The most up and down regulated genes were additionally evaluated using Pharos software^3^ for identification of molecules in clinical use that can down or up-regulate the identified genes.

Expression of bcl2 and tnf was quantified in group 4 animals. cDNA from the control, noise exposed and noise + MSC samples was synthesized using the miScript II reverse transcription kit (Qiagen, cat#218161). Semi quantitative PCR for bcl-2 and tnf (Origene cat MP201255; NM013693) was then performed on the Bio-Rad CFX using the Quantitect SYBR Green PCR kit (Qiagen, cat# 204141). Tranthyretin expression was measured using the Bio-Rad qMMuCID0006861 primer pairs.

All reactions were performed in triplicate and the Cq value was determined using the threshold calculated by the Bio-Rad software. Fold change was calculated using the 2^–ΔΔCT^ method.

### Histological Analysis

Group 3 animals were anesthetized with Beuthanasia (Merck Animal Health) 14 days after treatment with MSCs or artificial perilymph and fixed via intracardiac perfusion with 4% paraformaldehyde in PBS. The cochleae were removed and postfixed in 2.5% glutaraldehyde, 1.5% paraformaldehyde in 0.1 M phosphate buffer overnight at 4°C. Cochleae were decalcified in 1% glutaraldehyde in 0.12 M ethylene diamine tetraacetic acid (ETDA) in 0.1 M phosphate buffer for 4 days at 4°C. The specimen were then stained with osmium 1% for 1 h. After washing in distilled water specimens were dehydrated in a graded series of ethanol. After 30 min in propylene oxide (obtained from Electron Microscopy Sciences, Hatfield, PA, United States #20401), cochleae were transferred to araldite/P mix (obtained from Electron Microscopy Sciences, Hatfield, PA, United States #10900) and kept on a rotating shaker overnight. Specimen were then embedded in araldite in a vacuum oven. The blocks were baked in a 60°C oven. Serial sections at 30 μm were performed and stained with toluidine blue. After air drying, sections were mounted on slides with Permount for microscopic analysis.

Fog group 4 animals, the temporal bones were removed, the stapes extracted, and the round window was opened. The temporal bones were postfixed overnight in 4% paraformaldehyde in PBS at 4°C. After rinsing in PBS three times for 30 min, the temporal bones were decalcified in 10% ETDA for 48 h. The temporal bones were rinsed in PBS, dehydrated, and embedded in paraffin. Ten μm sections were cut in parallel to the modiolus, mounted on Fisherbrand^®^ Superfrost^®^/Plus Microscope Slides (Fisher Scientific, Pittsburgh, PA, United States) and dried overnight. Samples were deparaffinized and rehydrated in PBS two times for 5 min, then three times in 0.2% Triton X-100 in PBS for 5 min and finally in blocking solution 0.2% Triton X-100 in PBS with 10% FBS for 30 min at room temperature. Sections underwent antigen retrieval in a microwave using 1:10 Dako^®^ Target Retrieval Solution (DAKO Corporation, Carpinteria, CA, United States) for 30 s. Immunohistochemistry was carried out with anti-Bcl-2 rabbit polyclonal antibody diluted 1:200 (BD Biosciences, Inc., San Jose, CA, United States). The tissue was incubated overnight at 4°C in a humid chamber. After three rinses in PBS, immunohistochemical detection was carried out with an anti-Rabbit IgG (1:50; Vectastain Elite ABC Kit of Vector Laboratories, Inc., Burlingame, CA, United States). The secondary antibody incubated for 3 h at room temperature in a humid chamber and reactions carried out per manufacturer instructions. For TNFα immunohistochemistry the tissue was incubated for 48 h at 4°C in a humid chamber in TNFα polyclonal antibody (1:500, Thermo Fisher, PA5-19810). After three rinses in 0.2% Triton X-100 in PBS, immunohistochemical detection was carried out with an Alexa Fluor Donkey 555 anti-Rabbit (1:1000; Abcam). The secondary antibody was incubated for 6 h at room temperature in a humid chamber. The slides were rinsed in 0.2% Triton X-100 in PBS three times for 5 min and finally cover slipped with ProLong^®^ Gold antifade reagent (Invitrogen^TM^ Molecular Probes, Eugene, OR, United States). For detection of Bcl-2 expression, sections underwent antigen retrieval in a microwave using 1:10 Dako^®^ Target Retrieval Solution (DAKO Corporation, Carpinteria, CA, United States) for 30 s. Immunohistochemistry was carried out with anti-human Bcl-2 rabbit polyclonal antibody diluted 1:200 (BD Biosciences, Inc., San Jose, CA, United States). The tissue was incubated overnight at 4°C in a humid chamber. After three rinses in PBS, immunohistochemical detection was carried out with an anti-Rabbit IgG (1:50; Vectastain Elite ABC Kit of Vector Laboratories, Inc., Burlingame, CA, United States). The secondary antibody incubated for 3 h at room temperature in a humid chamber.

### Statistical Analysis

Statistical analysis of hearing outcomes was performed with ANOVA for repeated measures. RT PCR results were analyzed using ANOVA (Prism V8).

## Results

### Delivery of Mesenchymal Stromal Cells to the Mouse Cochlea Does Not Induce Hearing Loss

**FIGURE 1:**
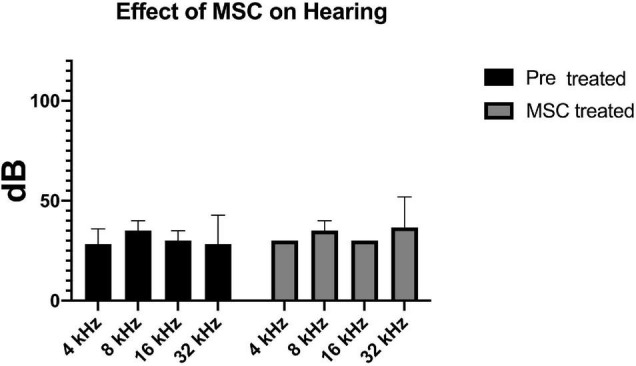
Delivery of MSCs did not have any effect on baseline hearing. Hearing thresholds measured by ABR at four different frequencies prior to (black bars) and 7 days after transplantation of MSC to the inner ear via the posterior semicircular canal (gray bars) are depicted. There is no significant difference between the pre- and post-transplantation thresholds.

After documentation of baseline hearing by ABR, 1 month old C57Bl/6 mice (*n* = 5) underwent delivery of 1 μl of human Wharton’s Jelly-derived MSCs into their left posterior semi-circular canal. One week post-delivery of cells, their hearing was re-evaluated. ABR recording prior to treatment at 4, 8, 16, and 32 kHz showed an average hearing threshold of 28.3 ± 7, 35 ± 5, 30 ± 5, and 30 ± 17, respectively. The average hearing threshold for each frequency did not change significantly after cell therapy (30 ± 0, 35 ± 5, 30 ± 0, and 40 ± 17 for 4, 8, 16, and 32 kHz, respectively) (Figure 1).

### Delivery of Mesenchymal Stromal Cells to the Mouse Inner Ear Provides Significant Hearing Rescue After Severe Noise Trauma

**FIGURE 2:**
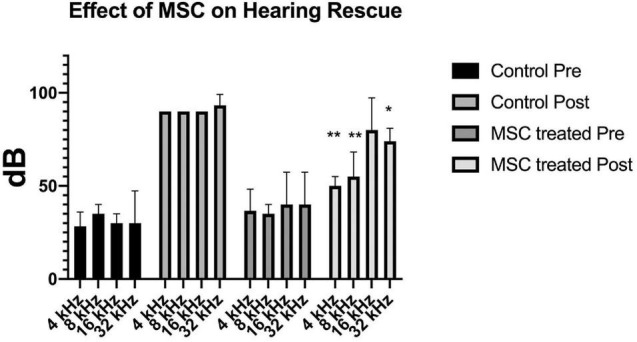
Delivery of MSCs rescue hearing after noise exposure. Exposure to sound for 4 h results in a profound hearing loss across all frequencies (medium gray bars; Control Post). Delivery of MSCs into the posterior semicircular canal of animals after sound exposure resulted in significant rescue of hearing at 4 and 8 kHz (*p* < 0.01**) as well as at 32 kHz (*p* < 0.05*) (light gray bars; MSC treated Post) when compared to the control animals treated with artificial perilymph (Control Post).

**FIGURE 3:**
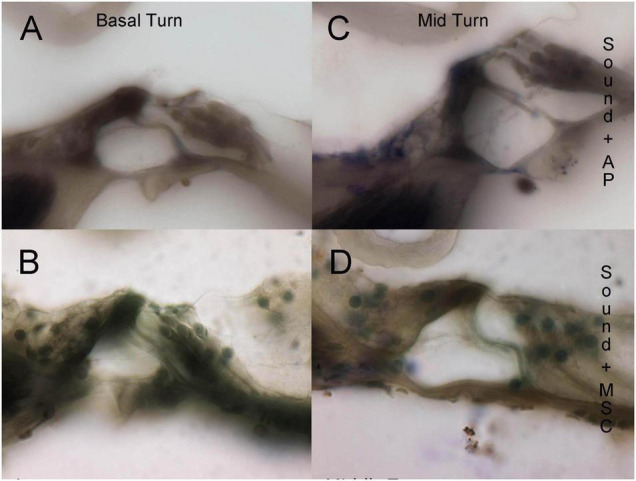
Histological evaluation of organ of Corti integrity in sound trauma **(A,C)** and sound trauma and MSC **(B,D)** treated cochlea. Loss of inner hair cells is consistently seen in the basal turns of both groups **(A,B)**. The MSC treated group showed occasional survival of inner hair cells at the midturn **(D).**

Pre-treatment ABR measurements showed normal hearing threshold in both groups (Figure 2; control ore and MSC treated pre). After noise exposure, animals of the control group (treated with artificial perilymph) demonstrated a severe to profound hearing loss across all frequencies at 14 days post-sound exposure (Figure 2; control post). There was a significant rescue effect seen in the MSC treated animals at 4 and 8 kHz (*p* < 0.01) and at 32 kHz (*p* < 0.05) (Figure 2; MSC treated post) when compared to the thresholds of the control group after noise exposure and application of artificial perilymph (Figure 2; control post). At 16 kHz, treated animals showed better hearing thresholds than sound trauma and artificial perilymph treated animals, but this was not statistically significant. Histological analysis of the organ of Corti performed separately for the basal and mid turn showed loss of hair cells both inner and outer hair cells for both groups after sound trauma (MSCs and artificial perilymph treated) in the basal turn (Figures 3A,B). When compared to the artificial perilymph treated animals (Figure 3C), the MSC-treated animals did show preservation in the mid turn of the cochlea (Figure 3D). DPOAEs were not measurable after sound trauma and were not restored by MSC therapy (data not shown).

### Delivery of Mesenchymal Stromal Cell Alters Cochlear Gene Expression

For control cochleae, an average of 155 ng/μl of RNA and for MSC treated cochleae 182 ng/μl of RNA were extracted. An average of 13,789,205 and 13,472,844 input reads for control and MSC treated cochleae were performed yielding 15,671 mapped genes. Delivery of MSCs caused a greater down-regulatory than up-regulatory effect on gene expression in sound traumatized animals treated with MSCs. A total of 2742 genes were down-regulated, and 1138 genes were found to be up-regulated. Significantly changed genes were considered to have an FDR of <0.05 and two fold change (up or down). Key down-regulated genes are shown in Supplementary Table 1 and up-regulated genes are shown in Supplementary Table 2.

### Analysis of Gene Ontologies and Functional Networks

**FIGURE 4:**
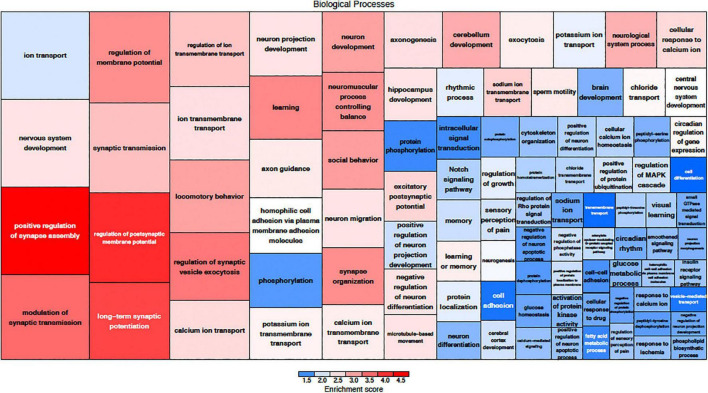
Down-regulated biological processes classified by gene ontology. The size of the category corresponds to a lower FDR with the more deeply red groups representing a higher enrichment score. The graphic representation of gene expression demonstrates that a key element in MSC treatment is modulation of synaptic assembly which is represented in multiple groups of down-regulated genes.

**FIGURE 5:**
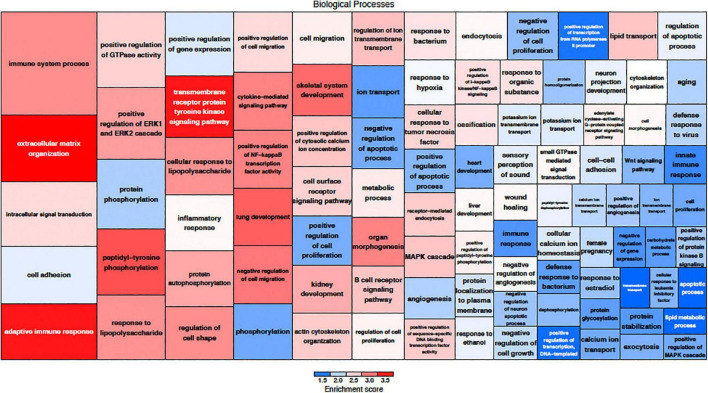
Up-regulated biological processes classified by gene ontology. The size of the category corresponds to a lower FDR with the more deeply red groups representing a higher enrichment score. Up-regulation of genes related to immune function are a key component of MSC treatment.

The effects of MSC delivery after sound trauma were evaluated using GO expression analysis using IPA “Core” and “Bioprofiler” analysis. The GO classification for genes that are regulated by MSC delivery were broken down into the biological processes, molecular functions and cellular components and weighted according to their enrichment (FPKM) and FDR score. These were also visualized as a Voronoi tree plots to facilitate identification of key pathways. Down-regulated genes were enriched for processes related to neuronal function and repair with ion transport, nervous system transport, positive regulation of synapse assembly, modulation of synaptic transmission, regulation of membrane potential, synaptic transmission, regulation of post-synaptic membrane potential and long-term synaptic potentiation GO classifications dominating (Figure 4). The GO molecular function classification showed significant changes in genes related to protein binding although these were not highly enriched. Genes related to transmitter-gated ion channel activity, voltage gated ion activity, syntaxin-1 binding, sodium channel activity and calcium channel activity were found. Interestingly, processes relating to dynein light and intermediate chain binding were also seen. The GO terms relating to cellular components concentrated on the excitatory synapse, components of the axon, dendrites, and myelin sheath as well as mitochondrion related annotations (Figure 5). Voronoi tree-maps that improved hierarchical representation of the GOEA data were additionally evaluated and groups of genes that appear significantly represented were identified (Bernhardt et al., 2009).

Down-regulated genes included miRNAs such as miR-9, miR-124a, miR-124-2, and miR-133a. Regulation of heat shock proteins has been implicated in sound trauma and in this data set, delivery of MSCs resulted in down-regulation of Hspa1b, Hsp1l, Hsph1, Ahsa1, Hsp9, and Hsp90ab1 (Gratton et al., 2011). Key effectors of cell death were also down-regulated whereas protective genes such as bcl-2 were up-regulated.

**FIGURE 6:**
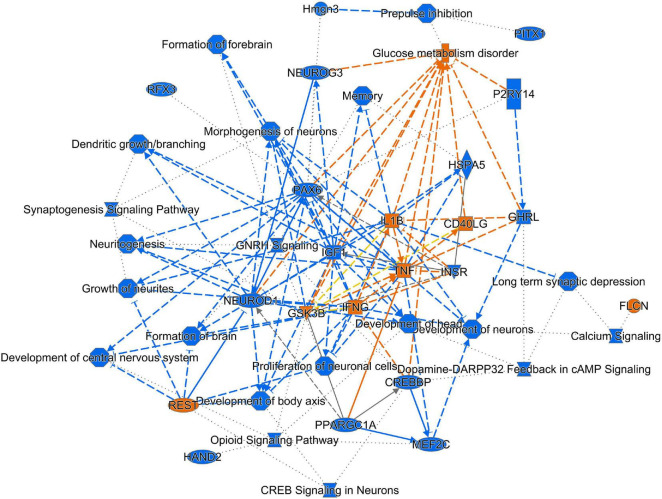
Graphical representation of IPA analysis of up- and down-regulated genes and processes. Using IPA core analysis of down-regulated genes and processes (blue) predicted up-regulation of genes and processes (orange). There is an overall down-regulation of pathways related to synaptogenesis, development of neurons and excitatory signaling. TNFα is a key factor that influences multiple processes and genes.

**FIGURE 7:**
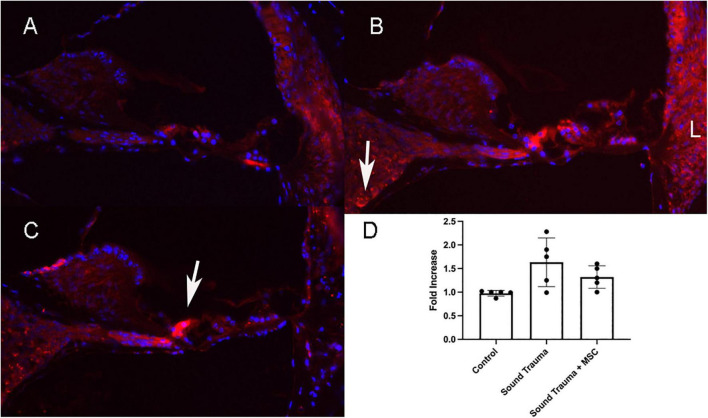
Expression of TNFα protein (Red **A–C**) and semi quantitative RT PCR of TNFα mRNA **(D)**. Control cochlea showed minimal TNF expression in the hair cells and lateral wall **(A)**. After sound trauma there was a clear increase in TFN alpha immunolabeling in hair cells, the spiral ganglion (arrow) and the lateral wall **(B)**. Treatment with MSCs after sound trauma reduced TNFα expression in the lateral wall and outer hair cells but expression remained high in the inner hair cell (arrow **C**). Semi quantitative RT-PCR showed an up-regulation of TNF gene expression after sound trauma. This was slightly reduced after MSC treatment but not to control levels **(D)**.

**FIGURE 8:**
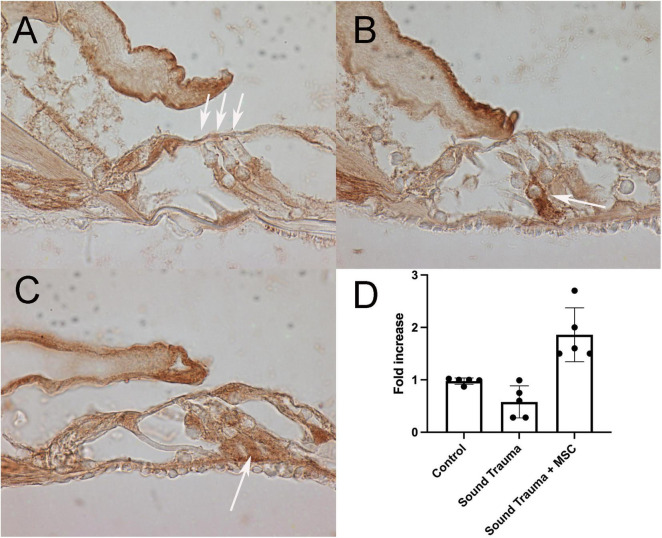
Expression of bcl-2 (brown **A–C**) and semi quantitative RT PCR of bcl-2 mRNA **(D)**. Control cochlea showed bcl-2 expression in the pillar, in the inferior portion of the outer hair cells (arrows) and neurites **(A)**. After sound trauma there was an occasional increase in bcl-2 immunolabeling in isolated supporting cells (arrow) **(B)**. Treatment with MSCs after sound trauma increased expression of bcl-2 in supporting cells (arrow **C**). Semi quantitative RT-PCR showed reduction of bcl-2 mRNA expression after sound trauma. Treatment with MSC after sound trauma showed a significant up-regulation of bcl-2 mRNA compared to both controls and sound trauma only treated animals (*p* = 0.003) **(D)**.

**FIGURE 9:**
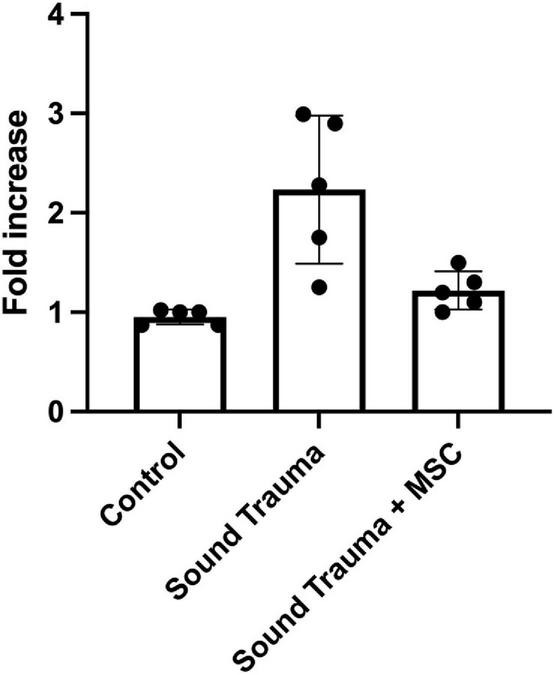
Expression of cochlear transthyretin by semi quantitative RT-PCR. Sound trauma treated animals showed a relative increase in expression of transthyretin mRNA. MSC treatment resulted in a significant reduction in expression of transthyretin mRNA after sound trauma (*p* = 0.001).

Up-regulated genes were enriched for processes involved in adaptive and innate immune responses, extracellular matrix organization, cell adhesion and a wide range of cell repair and developmental processes (Table 1). Additional processes included transcription factor function, wound healing, regulation of apoptotic process and response to hypoxia. Molecular function annotations centered on a range of protein receptor signaling processes and processes related to matrix binding. Signaling processes related to insulin like growth factor binding, TGFβ, fibroblast growth factor and Wnt binding were represented. Annotations showed that these genes were related to the cell surface, the extracellular matrix and the Golgi apparatus. Processes involving membrane repair and transmembrane tyrosine kinase signaling were also represented. Also represented were processes such as negative regulation of neuronal apoptosis, protein stabilization and sensory response to sound, which we would expect to see in a repair process involving the inner ear. Up-regulated genes related to response to sound are specific to the ear, associated with development of the inner ear, associated with syndromes that include hearing loss or when mutated are associated with sensorineural hearing loss (Shearer et al., 1993). We identified 14 up-regulated genes including Myo1A (Kwon et al., 2014), Ripor2 (Diaz-Horta et al., 2018), Nipbl, Ccdc50 (Modamio-Høybjør et al., 2007), Lhfpl5 (Ge et al., 2018), Fgfr1 (Pirvola et al., 2002), Diaph1 (Neuhaus et al., 2017), Pou4f3 (Walters et al., 2017), P2rx2 (Mittal et al., 2016), Otog (Schraders et al., 2012), Otoa (Lukashkin et al., 2012), Cntn5, Icam1 (Schmutzhard et al., 2011), and Hexa. The identified genes ranged from hair cell, spiral ganglion and stria specific to affecting tectorial membrane function, emphasizing that the MSC treatment affects multiple areas of the inner ear. Treatment with MSC resulted in both up and down regulation of genes that have been associated with noise induced hearing loss in other studies. Regulation of complement and toll receptor signaling has been shown to be associated with noise induced hearing loss (Patel et al., 2013; Vethanayagam et al., 2016; Yang et al., 2016). Members of the complement cascade identified included C1qtnf4, C1ql2, and Cfd (down-regulated) and C1rl and C3 (up-regulated). Other members of the innate immune system that showed differential expression were members of the Toll signaling system including Tlr4, Tlr6, and Tiarp, which were up-regulated. To more clearly identify effects of cell therapy the IPA software was used to compare only significantly up- and down-regulated genes to evaluate the relationship between expression data and potential pathways involved in injury and protection. Key canonical pathways identified were calcium signaling, synaptogenesis signaling, endocaniboid neuronal synapse pathway, GABA receptor signaling and CREB signaling in neurons. Key predicted upstream regulators include BDNF, which has been shown to be produced by MSCs and has been identified as a mechanism of MSC protection of neurons in stroke models (Gu et al., 2014; Mukai et al., 2018). When graphically represented, TNFα can be seen as playing a central role in the predicted changes in biological function and signaling pathways (Figure 6). This was further evaluated using immunohistochemistry and semi quantitative PCR (Figures 7–9). Immunofluorescent labeling showed that MSC therapy reduced expression of TNFα protein in the lateral wall (Figures 7B vs. C), however expression was higher than controls in the inner hair cells of both sound trauma only (Figure 7B) and sound trauma + MSC treated animals (Figure 7C). RNAseq analysis demonstrated a 0.8 fold up-regulation of TNFα mRNA in MSC vs. sound trauma treated animals. Evaluation using RT-PCR demonstrated that both sound trauma only and sound trauma + MSC treated animals showed significantly up-regulated mRNA levels compared to controls (*p* = 0.0021; *p* = 0.0002) but were not significantly different from each other (*p* = 0.2517). IPA comparison analysis (Figure 6) also identified NeuroD1 as a significantly down-regulated transcription factor that is predicted to modulate multiple pathways related to neuronal growth and survival. Many of the survival effects are mediated through neurotrophin signaling. One of the downstream effects of neuroD signaling are alterations in the balance of bcl-2 and bax modulating apoptosis (Wy, 2013). MSCs have also been shown to modulate bcl-2 expression in a variety of injury models (Xiang et al., 2009; Gu et al., 2014). Our data showed that there was a 0.4 fold up-regulation of bcl-2 in MSC treated animals vs. sound only controls. There were no identifiable changes in bax expression in our data suggesting induction of an anti-apoptotic state. This was confirmed using immunohistochemistry and RT-PCR (Figure 8). MSC treated animals showed an increase of bcl-2 immunolabeling in the organ of Corti, particularly in the supporting cells (Figure 8C) and demonstrated an up-regulation of bcl-2 mRNA (Figure 8D). One of the most down-regulated genes identified in our study was transthyretin (5.377 fold decrease in expression after MSC treatment). Transthyretin plays multiple roles in the nervous system and is a key modulator of metabolism, which was predicted to be altered in the IPA comparison analysis (Figure 6). We confirmed down-regulation of transthyretin by semi quantitative RT-PCR (Figure 9). Finally, we identified molecules in clinical use that could modulate most significantly regulated genes, identifying 46 drugs that could potentially be used to study some of the effects induced by MSCs (Table 2). Transthyretin can be down-regulated using thyroxine hormone (T4). Based on the Pharos analysis, multiple modulators of GABA neurotransmission are available, raising the possibility of using small molecules to modulate this pathway.

**TABLE 1:** Down- and up-regulated canonical pathways.

**Down-regulated canonical pathways**	**Up-regulated canonical pathways**
Calcium Signaling	Cardiovascular Disease, Cardiovascular System Development and Function
Synaptogenesis Signaling Pathway	Cancer, Organismal Injury and Abnormalities
GABA Receptor Signaling	Cell-To-Cell Signaling and Interaction, Hematological System Development and Function, Inflammatory Response, Organismal Functions
Endocannabinoid Neuronal Synapse Pathway	Hematological System Development and Function, Immune Cell Trafficking, Inflammatory Response, Tissue Development
Oxidative Phosphorylation	Cancer, Hematological Disease, Immunological Disease, Organismal Injury and Abnormalities
Opioid Signaling Pathway	Connective Tissue Development and Function, Skeletal and Muscular System Development and Function, Tissue Development
Mitochondrial Dysfunction	Cancer, Gastrointestinal Disease, Organismal Injury and Abnormalities
GNRH Signaling	Cellular Function and Maintenance
Synaptic Long Term Depression	Cell-To-Cell Signaling and Interaction, Cellular Function and Maintenance, Inflammatory Response
Corticotropin Releasing Hormone Signaling	Cancer, Gastrointestinal Disease, Organismal Injury and Abnormalities

**TABLE 2:** Pharos analysis of genes showing greatest up- and down-regulation that can be modulated by small molecules in clinical use.

**Symbol**	**Gene name**	**Symbol**	**Gene name**
Cacna1a	Voltage-dependent calcium channel subunit alpha-1/alpha	Hrh3	Histamine H3 receptor
Cacna1i	Voltage-dependent T-type calcium channel subunit alpha	Htr2c	5-hydroxytryptamine receptor 2C
Car4	Carbonic anhydrase 4	Irs4	Insulin receptor substrate 4
Cck	Cholecystokinin	Kcnd2	Potassium voltage-gated channel subfamily D member 2
Ccl3	C-C motif chemokine 3	Kcnh6	Potassium voltage-gated channel, subfamily H, member 6
Cnr1	Cannabinoid receptor 1	Kcns2	Potassium voltage-gated channel subfamily S member 2
Gabra1	Gamma-aminobutyric acid receptor subunit alpha-1	Krt1	Keratin, type II cytoskeletal 1
Gabra4	Gamma-aminobutyric acid receptor subunit alpha-4	Lipf	Gastric triacylglycerol lipase
Gabra6	Gamma-aminobutyric acid receptor subunit alpha-6	Muc1	Mucin 1, transmembrane
Gabrb1	Gamma-aminobutyric acid receptor subunit beta-1	Ngf	Beta-nerve growth factor
Gabrb2	Gamma-aminobutyric acid receptor subunit beta-2	Pou1f1	Pituitary-specific positive transcription factor 1
Gabrp	Gamma-aminobutyric acid receptor subunit pi	Prl	Prolactin
Gal	Galanin peptides	Prlr	Prolactin receptor
Gh	Growth hormone	Rnf112	RING finger protein 112
Gnrhr	Gonadotropin releasing hormone receptor	Scn2a1	Sodium channel protein
Gp2	Pancreatic secretory granule membrane major glycoprotein GP2	Slc5a1	SGLT1 protein
Gria1	Glutamate receptor 1	Slc6a1	Sodium- and chloride-dependent GABA transporter 1
Gria2	Glutamate receptor 2	Slc6a5	Sodium-dependent noradrenaline transporter
Grik2	Glutamate receptor ionotropic, kainate 2	Sstr3	Somatostatin receptor type 3
Grin2a	Glutamate receptor ionotropic, NMDA 2A; NMDA 2B; NMDA 2D	Stum	Protein stum homolog
Grin2b	Glutamate receptor ionotropic, NMDA 2B	Tnr	Tenascin-R
Grin2c	Glutamate receptor, ionotropic, NMDA2C	Trpm8	Transient receptor potential cation channel subfamily M member 8
Grm5	Metabotropic glutamate receptor 5	Ttr	Transthyretin

**FIGURE 10:**
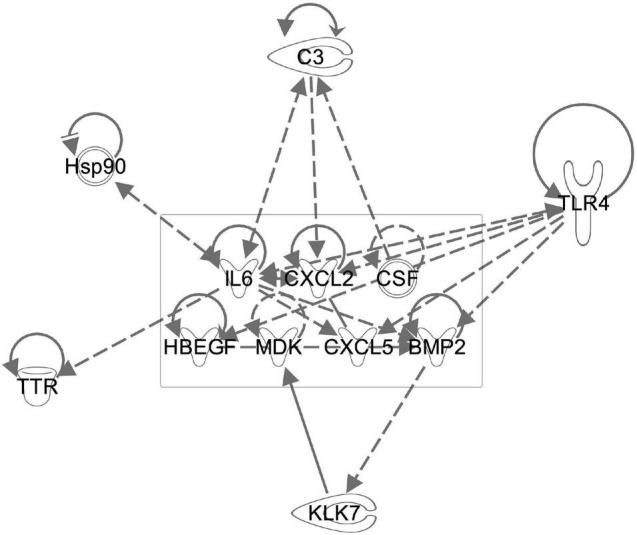
The known WJC secretome regulates multiple genes found to be altered after sound trauma and MSC delivery. Components of the WJC secretome (boxed area) were evaluated for potential interactions with genes that we found to undergo significant change using the IPA knowledgebase. This demonstrates the potential biological basis for the protective effects that WJCs have on noise trauma.

Besides neurotrophins, MSCs produce a variety of immunomodulatory molecules and other growth factors. Using the predictive functions of IPA, we analyzed the potential effect of several growth factors and cytokines known to be delivered by MSC on our data set and found that the observed changes in HSP90, complement factor 3 and toll like receptor 4 could potentially be modulated by MSCs (Figure 10).

## Discussion

Intra-labyrinthine application of WJCs after noise trauma showed significant protection of hearing threshold at the lower frequencies at 4 and 8 kHz and to a lesser degree at 32 kHz in mice. Histological changes related to sound trauma could be identified in the organ of Corti in both groups. There was preservation of some mid turn hair cells in the MSC treated group. The changes underlying the altered threshold could also be the result of damage to other regions, for example of the stria vascularis or the synapses. Since the focus of the present study was the functional restoration and RNA changes induced by cell therapy rather than the histological changes, synapses and the stria vascularis were not investigated. We identified more than 2700 down-regulated and 1138 up-regulated genes after treatment with WJC. To understand the biological effects of the differentially regulated genes, the cellular components, molecular function, and biological process were analyzed by GO annotation. Using IPA, specific analysis of genes related to cell death and survival showed the regulation of 365 molecules related to necrosis and 353 molecules related to apoptosis with a predictive effect of decreasing both death mechanisms and increasing cell survival. We could demonstrate that there are MSC induced reductions of TNFα expression in the cochlear lateral wall (Figure 7) and MSC induced increases in bcl-2 expression in the organ of Corti (Figure 8). TNFα up-regulation has been associated with sound trauma (Frye et al., 2019). Our data demonstrated an up-regulation in TNFα gene expression which could not be confirmed by PCR (Figure 7D). However, distribution of TNFα expression was reduced in the lateral wall of the cochlea (Figures 7C vs. B). Increased expression of TNFα in the sound damaged cochlea has been demonstrated for days 1–3 post-sound trauma followed by a decrease and then a further expression increase at 28 days post-noise exposure (Fujioka et al., 2006). Additional time points will be needed to evaluate the long term effects of MSC treatment. We additionally evaluated the effect of MSC treatment on bcl-2 expression in the sound damaged inner ear. Our RNAseq data demonstrated a down-regulation of cell death effector genes and an up-regulation of the antiapoptotic bcl-2 (Figure 8) consistent with other MSC experiments (Gu et al., 2014). Up-regulation of bcl-2 has been shown to be an underlying mechanism in sound conditioning protection from noise trauma (Niu et al., 2003).

Many of the identified genes and GO annotations have been found in or belong to a similar GO pattern seen in stroke or TBI suggesting that severe sound trauma and TBI (Spaethling et al., 2008; Darkazalli et al., 2017; Meng et al., 2017; Trivedi et al., 2019; Li et al., 2020) involve similar molecular mechanisms. By evaluating the diverse pathways activated in this recovering system, we may be able to select new targets for the pharmacological mitigation of sound trauma. Significant advances have been made in the pharmacologic treatment of brain injury and many of the drugs that are in experimental or clinical trials (Diaz-Arrastia et al., 2014) may also be applicable to the inner ear. Use of the Pharos database identified that 46 of the most up and down regulated genes could potentially be targeted by molecules that are already in use. NMDA receptors in particular have already been targeted in clinical trials for sound induced tinnitus, demonstrating that MSC induced protection in part functions through previously identified mechanisms (Bing et al., 2015; Staecker et al., 2017).

In the cellular component category, the most represented term is “AMPA glutamate receptor complex,” followed by “post-synaptic density membrane” and “excitatory and inhibitory synapses” for the down-regulated genes. In the molecular function category, the term “transmitter-gated ion channel activity involved in regulation of post-synaptic membrane potential” and “syntaxin-1 binding was found to be represented. The underlying biological processes in the down-regulated genes were related to synaptic functions. Most striking, synapse-specific processes like excitatory as well as inhibitory synapse, AMPA glutamate receptor complex synapse assembly, regulation of post-synaptic membrane potential, modulation of synaptic transmission and long-term synaptic potentiation were significantly down-regulated. This suggests that neuronal repair and control of excitotoxicity could be key components of recovery from severe sound trauma. For the up-regulated genes, “*trans*-Golgi network transport vesicles” is the most over-represented term in the cellular component category. Looking at molecular functions, extracellular binding, Wnt-protein and insulin-like growth factor binding were most abundant alongside with cytokine receptor activity. In the biological processes category, the terms extracellular matrix organization, adaptive immune response as well as transmembrane receptor protein tyrosine kinase (Trk) signaling pathway were found to be strongly represented. Immunomodulation and activation of Trk signaling pathways are known to be potent enhancers of survival. For example, systemic transplantation of MSC in a TBI model led to a lower density of microglia, macrophages as well as peripheral infiltrating leukocytes at the injury site, to reduced levels of pro-inflammatory cytokines and increased anti-inflammatory cytokines (Zhang et al., 2013). Even in a model of xenotransplantation of MSC derived from adipose tissue after ventral root avulsion, an increased neuronal survival and partial preservation of synaptophysin-positive nerve terminals as well as a reduction of pro-inflammatory reactions were elicited (Ribeiro et al., 2015). It is assumed that by suppressing lymphocyte, astroglia and microglia effects, MSC can prevent a second delayed neuronal injury (Ribeiro et al., 2015). Furthermore, MSCs can prevent neuronal cell death not only via restoration of the local microenvironment but also by the release of classic inhibitors of apoptosis (Tran and Damaser, 2014).

Looking at the top down-regulated genes, mainly genes coding for transcription factors or for proteins and receptors related to synaptic function are among the most regulated. Down-regulation of oxidative stress pathways might be key for the mediation of protective effects. According to our RNA sequencing data, transthyretin (Ttr), a neuronal stress marker, is one of the most significantly down-regulated genes by the application of MSC after noise-induced trauma. It has been shown that Ttr level correlate with ROS or reactive nitrogen species (RNS) (Saito et al., 2005; Fong and Vieira, 2013). In addition, the expression of the Ttr gene is regulated by glucocorticoids, which are stress hormones (Li et al., 2011; Martinho et al., 2012). On a protein level, Ttr can initiate oxidative stress in the endoplasmic reticulum and is thereby involved in the unfolded protein response (UPR) (Teixeira et al., 2006; Genereux et al., 2015; Chen J. et al., 2016). Thus, down-regulation of ttr might result in inhibition of formation of reactive oxygen and nitrogen species thereby reducing the extent of trauma-associated damage. Recent TBI studies using drop-seq have demonstrated that up-regulation of ttr is one of the major markers of trauma in a wide range of cell types (Arneson et al., 2018). This study also documented functional recovery induced by dosing TBI animals with thyroxine (T4) which down-regulates transthyretin. We demonstrated an MSC induced normalization of ttr gene expression by RNAseq and semiquantitative PCR (Figure 9) raising the possibility that thyroxine could be used to limit sound trauma induced damage. Other factors involved in the production of ROS belong to the kallikrein-kinin system. This system includes plasma or tissue related kinins and they mediate vascular permeability, edema formation, inflammation and the release of glutamate in the case of neurodegenerative disease associated with neuroinflammation such as TBI or Alzheimer’s disease (Naffah-Mazzacoratti Mda et al., 2014; Hopp and AlbertWeissenberger, 2015). Tissue-derived kallikreins belong to the chymotrypsin- and trypsin-like serine endopeptidases. Especially kallikrein-7, originally identified as an inflammation−induced proteolytic enzyme in the skin, is down regulated by the application of WJS after sound trauma (Kidana et al., 2018). In addition, stress-related heat shock proteins that are involved in the response to stress and metabolic damage to the inner ear such as Hspa1b, Hsp1l, Hsph1, Ahsa1, Hsp9, and Hsp90ab1 are also down-regulated by MSC treatment (Sheehan-Rooney et al., 2013).

Moreover, several transcription factors, e.g., BarHl2, Neurod2, EN2, and Pax6, are down-regulated by application of MSC in noise trauma as demonstrated by our results. The homeobox protein BarH-like 2 (Barhl2) acts as transcription factor and possesses an enhancer that can be activated by the basic helix-loop-helix factor Atoh1 and can drive spinal cord-specific gene expression. Interestingly, this gene is significantly down-regulated when comparing noise-damaged cochlea treated with MSC to the sham-treated. Similar is observed for the neuronal differentiation factor 2, Pax6 and homeobox protein engrailed-2 (EN2). The later may alter the excitation/inhibition balance, and its down-regulation may rescue or regain homeostasis by MSC to reduce damage. NeuroD2 and Pax6 are important regulators of neuronal development in the central nervous system and their down-regulation after treatment with MSC seems quite surprising (Yogarajah et al., 2016). Whether damage leads to dedifferentiation and induction of neurogenesis thereby making the inner ear more susceptible to secondary damage mediated by oxidative stress and therewith associated metabolites remains speculative and needs further investigation. In addition to neuronal development, NeuroD2 balances synaptic neurotransmission and intrinsic excitability in the adult system, thereby decreasing neuronal excitability of cortical pyramidal neurons both *in vitro* and *in vivo* (Chen F. et al., 2016). After trauma, as has been shown for example in models of TBI, there is an excitation/inhibition imbalance as also reflected by the GABA/glutamate imbalance. Excitatory pathways in the brain are regulated by GABA and loss of GABA releasing cells after trauma further enhances cell injury and apoptosis (Guerriero et al., 2015). Although glutamate is the excitatory transmitter in the brain required for proper function, post-traumatic burst release of glutamate leads to excitotoxicity and subsequent cellular injury as has been also shown after noise-induced damage of the inner ear. By down-regulation of both, glutamate and GABA receptor subunits, MSC might down regulate the neuronal activity thereby possibly preventing further damage and transforming the cochlea to a state for protection and regeneration. The availability of multiple small molecules modulating this pathway (Table 2) raises the possibility of novel treatments of sound trauma.

Several miRNAs (miR-9, miR-124a, miR-124-2, and miR-133a) are also down-regulated after treatment with MSCs. These miRNAs play a variety of important developmental roles; miRNA-9 is the most significantly up-regulated miRNA in the developing brain, targeting the transcription factors FoxG1, Hes1, or Tlx, thereby playing an important role in the regulation of neuronal progenitors (Coolen et al., 2013). MicroRNA-9 has also been shown to regulate Schwann cell migration in neuronal injury, and is thought to play an important role in peripheral neuronal repair (Zhou et al., 2014). Similarly, miRNA-124a is an important developmental regulator and is involved in neurogenesis of the hippocampus and retinal cones (Sanuki et al., 2011). This miRNA has also been shown to be up-regulated in repetitive brain injury (Huang et al., 2018). Therefore, the down-regulation of miR-124 observed in this study may represent a positive effect of MSC treatment. Both miR-9 and -124 have been identified as biomarkers of TBI (Ji et al., 2016). Thus, MSCs regulate not only transcription factors involved in neurogenesis, but also miRNAs involved in the regulation of transcription factors. Another miRNA that we found to be down-regulated (miR-133) is involved in organogenesis, regulating the hedgehog pathway in myogenesis (Mok et al., 2018; Yu et al., 2019). This miRNA has been found to protect neurons when up-regulated but is also involved in the remodeling of connective tissue after injury (Xin et al., 2012; Li et al., 2018). To fully understand the relevance of the changes induced by MSC in the inner ear, investigation of further time points after damage and treatment or even transcriptomics at single cell resolution will be needed.

The biological effects of MSCs have been widely studied. MSCs produce a wide range of growth factors, extracellular vesicles and miRNAs that taken as a whole have a wide range of potential effects on a tissue. The secretome of umbilical cord MSCs has been shown to contain Ntf3, Egf, Mdk, Hbegf, Cxcl5, Cxcl2, and Fgf9 (Hsieh et al., 2013). Interestingly, many of these factors are involved in neurogenesis and *in vitro*, they up-regulate a variety of transcription factors that enhance local release of some of the same growth factors, thereby amplifying the repair process. Many of the regulated genes we also identified in this study. Immunomodulation is also a well characterized feature of MSCs (Hsieh et al., 2013). We evaluated the potential effect of some of these reported growth factors by modeling their regulation of genes we saw change using IPA software. As depicted in Figure 10, the IPA knowledge base predicts that growth factors produced by WJCs can regulate factors such as C3, Tlr4, bcl2, and Ttr that we identified. Fully characterizing the links between MSC signaling and our findings is difficult since interactions of MSCs with their target tissue may actually alter their secretome (Drago et al., 2013). Evaluation of extracellular vesicles produced by WJCs for the mitigation of sound trauma has demonstrated that similar protective effects can be achieved using only the secretome alone (Warnecke et al., 2020).

Several limitations are associated with the present study. The study was focused on identifying the effects of MSC cell therapy on the cochlear transcriptome after noise exposure. Further insights could be gained by including analysis of the transcriptome from a group of mice not exposed to noise with and without treatment with MSC. Sound trauma-induced changes in gene expression have been evaluated in numerous publications (Cai et al., 2012; Patel et al., 2013). We chose a severe form of sound trauma to test the ability of WJC type MSCs to rescue an inner ear function. The identified pathways are similar to the ones seen in other MSC-based rescue of models and in analysis of TBI induced neuronal injury. To further characterize these effects, a more moderate sound trauma paradigm with multiple sample time points after MSC delivery and verification of the effects using PCR and immunohistochemistry will be needed. Despite these limitations, main cell signaling pathways involved in MSC-mediated inner ear protection after noise trauma have been identified by applying pathway enrichment analysis. In future studies, we could mimic the effects of MSCs or inhibit potential protective pathways with small molecules to better characterize regulatory pathways by using large-scale transcriptome analysis in well-defined sets of experiments (Islam et al., 2019).

## Conclusion

Delivery of WJC type MSCs after sound trauma has a significant rescue effect on hearing. RNAseq analysis demonstrates regulatory changes in multiple genes including genes related to excitatory neurotransmission and the innate immune system. Further studies using different sound trauma paradigms and different timing of cell delivery as well as additional replicates are needed to better characterize the effects mediated by MSCs in the present study.

## Data Availability Statement

The original contributions presented in the study are publicly available. The data presented in the study are deposited in the NCBI GEO repository, accession number GSE187029: https://www.ncbi.nlm.nih.gov/geo/query/acc.cgi?acc=GSE187029.

## Ethics Statement

The studies involving human participants were reviewed and approved by University of Kansas Human Subjects Committee (KU-Lawrence IRB approval #15402) and Stormont-Vail (SMV) Hospital (SMV IRB Approval conferred by IRB Chair, Jo-Ann S. Harris, MD). The patients/participants provided their written informed consent to participate in this study. The animal study was reviewed and approved by University of Kansas Health System, Institutional Animal Care and Use Board Committee.

## Author Contributions

AW and HS research design, data analysis, and writing of the manuscript. JH and MD data analysis and writing of the manuscript. MS electrophysiology and manuscript approval. AM preparation and delivery of MSC and manuscript approval. IM bioinformatics and manuscript approval. All authors contributed to the article and approved the submitted version.

## Conflict of Interest

HS is a consultant for MedEl GmbH and Otonomy Inc. and holds shares in Rescue Hearing Inc. AM is a shareholder in Ronawk Inc. The remaining authors declare that the research was conducted in the absence of any commercial or financial relationships that could be construed as a potential conflict of interest.

## Publisher’s Note

All claims expressed in this article are solely those of the authors and do not necessarily represent those of their affiliated organizations, or those of the publisher, the editors and the reviewers. Any product that may be evaluated in this article, or claim that may be made by its manufacturer, is not guaranteed or endorsed by the publisher.

## Footnotes

1
sequentiabiotech.com

2
reactome.org

3
Pharos.nih.gov

## Supplementary Material

Supplementary Table 1 Genes down-regulated in response to sound trauma followed by MSC treatment.

Supplementary Table 2 Genes up-regulated in response to sound trauma followed by MSC treatment.
